# COVID-19 Vaccine-Associated Transient Global Amnesia: A Disproportionality Analysis of the WHO Safety Database

**DOI:** 10.3389/fphar.2022.909412

**Published:** 2022-05-20

**Authors:** Diane Merino, Alexandre O. Gérard, Elise K. Van Obberghen, Nouha Ben Othman, Eric Ettore, Bruno Giordana, Delphine Viard, Fanny Rocher, Alexandre Destere, Michel Benoit, Milou-Daniel Drici

**Affiliations:** ^1^ Department of Psychiatry, University Hospital of Nice, Nice, France; ^2^ Department of Pharmacology and Pharmacovigilance Center of Nice, University Hospital of Nice, Nice, France; ^3^ Department of Nephrology-Dialysis-Transplantation, University Hospital of Nice, Nice, France; ^4^ Department of Neurology, University Hospital of Nice, Nice, France

**Keywords:** COVID-19 vaccines, transient global amnesia, adverse drug reaction, clinical epidemiology, pharmacology

## Abstract

Coronavirus disease 2019 (COVID-19) spread rapidly, resulting in a global pandemic for which vaccines were quickly developed. As their safety continues to be monitored, cases of transient global amnesia (TGA) following mRNA vaccination with elasomeran have been reported. TGA is characterized by sudden onset of anterograde amnesia with preservation of other cognitive functions and resolution within 24 h. We aimed to investigate the potential link of TGA with COVID-19 vaccines. We queried the World Health Organization VigiBase^®^ for all reports of “Transient global amnesia”, up to 6 December 2021. Disproportionality analysis relied on the Reporting Odds Ratio (ROR) with its 95% Confidence Interval (CI) and the Information Component (IC). A positive lower end of the 95% CI of the IC (IC025) is used to statistically detect a signal. Of all TGA cases, 289 were associated with a COVID-19 vaccine, representing the most frequent association. Tozinameran was mostly represented (147, 50.8%), followed by AZD1222 (69, 23,8%), elasomeran (60, 20.8%), and JNJ-78436735 (12, 4.2%). With an IC025 > 0, COVID-19 vaccines showed a significant ROR (5.1; 95%CI 4.4–6.0). Tozinameran reached the strongest ROR (4.6; 95%CI 3.9–5.0), followed by elasomeran (4.4; 95%CI 3.4–6.0), AZD1222 (3.8; 95%CI 3.0–5.0), and JNJ-78436735 (3.7; 95%CI 2.1–6.0). Our analysis of COVID-19 vaccines-related TGA reports shows significant disproportionality. Cerebrovascular, inflammatory, or migrainous mechanisms may underlie this association. Yet, numerous confounding factors cannot be tackled with this approach, and causality cannot be ascertained. The identification of this trigger of TGA may help the clinician in his etiological research.

## Introduction

At the end of 2019, in China, a cluster of viral pneumonia cases developed, later identified as COVID-19 (Coronavirus disease 2019). It spread rapidly, resulting in a global pandemic for which specific vaccines were soon developed. Indeed, by the end of 2020, several vaccines became available. Their rapid development was driven by previous experience with various vaccine platforms, investments in new technologies and an accelerated process of efficacy studies. (Bok et al., 2021). As the safety of authorized vaccines continues to be closely monitored, a signal emerged recently regarding five cases of transient global amnesia following mRNA vaccination with elasomeran. (ANSM, 2021).

Transient global amnesia (TGA) is a clinical syndrome characterized by the sudden onset of anterograde amnesia (the inability to form new memories) with some degree of retrograde amnesia. Other cognitive functions are preserved and TGA episodes resolve spontaneously within 24 h (Arena and Rabinstein, 2015). In 1990, relevant specific criteria enabled a standardized assessment of TGA. (Hodges and Warlow, 1990). With an incidence ranging between three and eight per 1,00 ,000 people per year, TGA mostly affects people aged 50–70 years (Bartsch and Deuschl, 2010) with a possible influence of gender. (Quinette et al., 2006). The pathophysiology of TGA remains unclear, although several hypotheses are being explored, such as cerebrovascular, migrainous and epileptic mechanisms (Szabo, 2014). An increased incidence of TGA has been suggested since the beginning of the COVID-19 pandemic, but still has to be confirmed, pertaining to COVID-19 infection itself or the stress induced by the constraints endured by the population. (Werner et al., 2020; Ramanathan and Wachsman, 2021; Hornick et al., 2022). Yet, the association of TGA with various drugs (e.g. phosphodiesterase inhibitors) has been previously documented. (Gandolfo et al., 2003; Schiefer and Sparing, 2005; Tsai et al., 2009; Larner, 2017). Therefore, we aimed to investigate the potential link of TGA with COVID-19 vaccines, relying on the international pharmacovigilance database. Since COVID-19 vaccines are subject to intense media coverage and a plethora of pharmacovigilance reports, the number of TGA reports may also artificially increase. To mitigate the impact of these possible confounding factors as much as possible, a disproportionality analysis has been conducted.

## Materials and Methods

### Data Source

Since 1978, the World Health Organization (WHO) mandates the Uppsala Monitoring Centre (UMC) to monitor drug safety worldwide. The UMC is an independent centre for drug safety and scientific research, which aims to collect evidence about harm to patients and to identify safety signals. (UMC, 2022). Data issued by each of the 172 national pharmacovigilance network members, as well as pharmaceutical laboratories, feed the UMC database. VigiBase^®^ (Lindquist, 2008) collects spontaneous reports, ensuring the preservation of the anonymity of both patients and notifiers.

Sociodemographic characteristics (age, sex, area of residence, notifier’s country), and details concerning the reported effect (suspected drugs, concomitant drugs, adverse drug reaction, date of occurrence, and seriousness (Gautron et al., 2018; U.S. Food and Drug Administration, 2022) are collected.

### Query

We queried VigiBase^®^ for all notified cases of TGA, irrespective of drug classes, from 14 November 1967 (first reports in VigiBase^®^) to 6 December 2021. Then, all Active Ingredients deemed suspect or interacting in four or more cases were ranked by absolute number of cases.

In practice, the TGA reports were defined by the Medical Dictionary for Regulatory Activities (MedDRA, version 24.1), with ‘transient global amnesia’ as a Preferred Term (PT). (MedDRA, 2021). A preferred term (PT) is the description that is considered to be the most clinically accurate to express a medical concept.

### Disproportionality Analysis

To mitigate the impact of potential overreporting of cases involving COVID-19 vaccines, a disproportionality analysis was conducted. We sought a potential pharmacovigilance signal relying on a disproportionality approach for all drugs. This disproportionality analysis was based on the Reporting Odds Ratio (ROR) and the Information Component (IC).

ROR is an equivalent of the odds-ratio (used in case-control studies), specific to case-non-case studies. It indicates the odds of a chosen adverse drug reaction (ADR) occurring with a specific active ingredient, relative to the odds of the same reaction being reported with all other drugs in the database. The precision of the approximate of ROR is reflected by a 95% Confidence Interval (CI). If the lower limit of the 95% CI of a drug’s ROR is > 1, a signal is suggested: the ADR is more frequently reported with this drug. Conversely, a ROR = 1 indicates an absence of signal: the ADR is reported similarly with the drug in comparison with other drugs. The higher the ROR, the more statistically relevant is the pharmacovigilance signal.

IC is used to evaluate the strength of the association between a given active ingredient and an ADR. It reduces the risk of false-positive signals, especially if the chosen ADR has a very low expected frequency in the database (artificially increasing the ROR). (Bate and Evans, 2009). A positive IC shows the superiority of the number of observed reports over the number of expected reports. The bottom end of the 95% CI of the Information Component is the IC025. For UMC, a positive IC025 is needed to statistically confirm the detection of a signal. (VigiBase, 2021).

In this disproportionality analysis, relevant associations were selected using IC025. Then, Active Ingredients were ranked and compared according to their association with TGA, through their ROR. As a subgroup analysis for COVID-19 vaccines reports, age and sex-specific disproportionality was assessed through calculation of the ROR and the IC025. As a sensitivity analysis for COVID-19 vaccines reports, disproportionality was calculated for physician-reported cases only. Characteristics of reports were described in terms of medians (with interquartile range) for quantitative variables, and in terms of effectives and proportions for qualitative ones.

## Results

### Characteristics of the Reports

As of 6 December 2021, 852 cases of transient global amnesia (PT) were collected in VigiBase^®^, 620 of which (72,8%) were deemed serious. (Gautron et al., 2018; U.S. Food and Drug Administration, 2022). Most cases concerned women, with 495 (58.1%) reports. The most represented age range was the 45–64 years group, with 286 (33.6%) reports, and the median age was 65 years. The median time to onset was 7 days (IQR 1–63.75). Almost half of the notifications came from the United States. When mentioned, the notifier was mostly a healthcare professional (46.5%).

Of all TGA cases, 289 were associated with a COVID-19 vaccine, which represented the most frequent association. Women were mostly involved, with 87 (64.7%) reports. The 45–64 years group was the most represented, with 101 (34.9%) reports, and a median age of 66 years. Among COVID-19 vaccine-related TGA cases, 178 (61.6%) were considered serious. The median time to onset for these cases was 5 days (IQR 1-15). Apart from amnesia, the most frequent co-reported MedDRA clinical terms in cases involving COVID-19 vaccine were confusional state in 27 reports (9.3%), headache in 31 reports (10.7%), and fatigue in 24 (8.3%) reports. Detailed characteristics of the reports are provided in Table 1.

**TABLE 1:** Characteristics of the reports of patients with TGA.

Characteristics	Number (%)	
	All cases	COVID-19 vaccines
Total	**852 (100)**	**289 (100)**
Sex		
Female	495 (58.1)	187 (64.7)
Male	337 (39.6)	101 (34.9)
Unknown	20 (2.3)	1 (0.3)
Age		
2–11 years	1 (0.1)	0
12–17 years	3 (0.4)	0
18–44 years	34 (4.0)	9 (3.1)
45–64 years	286 (33.6)	101 (34.9)
65–74 years	261 (30.6)	96 (33.2)
≥75 years	104 (12.2)	36 (12.5)
Unknown	163 (19.1)	47 (16.3)
Country		
United States of America	404 (47.4)	136 (47.1)
Germany	79 (9.3)	18 (6.2)
United Kingdom	65 (7.6)	34 (11.8)
Netherlands	44 (5.2)	27 (9.3)
Spain	38 (4.5)	14 (4.8)
France	36 (4.2)	12 (4.2)
Australia	34 (4.0)	14 (4.8)
Italy	33 (3.9)	6 (2.1)
Canada	26 (3.1)	0
Sweden	16 (1.9)	10 (3.5)
Switzerland	12 (1.4)	4 (1.4)
Austria	10 (1.2)	1 (0.3)
Japan	10 (1.2)	0
Portugal	7 (0.8)	2 (0.7)
Czechia	5 (0.6)	3 (1.0)
Denmark	5 (0.6)	1 (0.3)
Norway	5 (0.6)	2 (0.7)
Finland	4 (0.5)	2 (0.7)
Brazil	3 (0.4)	0
Ireland	3 (0.4)	2 (0.7)
Latvia	1 (0.1)	1 (0.3)
Unknown	13 (1.5)	0
Reporter qualification		
Healthcare Professional	396 (46.5)	58 (20.0)
Physician	277 (32.5)	48 (16.6)
Pharmacist	39 (4.6)	5 (1.7)
Other Health Professional	80 (9.4)	5 (1.7)
Others	272 (32.0)	87 (30.1)
Lawyer	4 (0.5)	0
Consumer/Non-Health Professional	268 (31.5)	87 (30.1)
Unknown	200 (23.5)	146 (50.5)

“Antiinfectives for systemic use” (ATC J), which comprises COVID vaccines, was the most represented drug class in the Anatomical Therapeutic Chemical (ATC) classification system, with 357 (42.0%) notifications. Among COVID-19 vaccines, tozinameran was mostly involved with 147 (50.8%) reports, followed by AZD1222 with 69 (23,8%) reports, elasomeran with 60 (20.8%) reports, and JNJ-78436735 with 12 (4.2%) reports.

Statins were reported in association with TGA, although less frequently than COVID-19 vaccines, with atorvastatin, simvastatin, and rosuvastatin, representing 41 (4.8%), 18 (2.1%), and 15 (1.8%) of the TGA reports, respectively. Influenza vaccines (inactivated) represented 23 (2, 7%) reports. Phosphodiesterase type 5 (PDE5) inhibitors, sildenafil and tadalafil, were each suspected in 17 (2.0%) reports. Further, 15 (1.8%) reports were associated with the hypnotic drug zolpidem (Table 2).

**TABLE 2:** Disproportionality analysis for reports of TGA.

Active ingredient	ROR	95% CI	Number of cases (%)
Tadalafil	19.3	11.9–31.0	17 (2.0)
Zolpidem	12.2	7.5–20.0	15 (1.8)
Atorvastatin	11.1	8.1–15.0	41 (4.8)
Sildenafil	9.9	6.1–16.0	17 (2.0)
Rosuvastatin	8.1	4.9–15.0	15 (1.8)
Simvastatin	8.1	5.1–13.0	18 (2.1)
Alendronic acid	7.1	3.9–13.0	11 (1.3)
Etoricoxib	6.9	2.6–18.0	4 (0.5)
Baclofen	6.9	3.3–14.0	7 (0.8)
Iodixanol	6.6	2.5–18.0	4 (0.5)
Ibandronic acid	5.2	2.0–14.0	4 (0.5)
COVID-19 vaccine	5.1	4.4–6.0	289 (33.9)^a^
Tozinameran	4.6	3.9–5.0	147 (50.8)
Elasomeran	4.4	3.4–6.0	60 (20.8)
AZD1222	3.8	3.0–5.0	69 (23.8)
JNJ 78436735	3.7	2.1–6.0	12 (4.2)
Heparin	4.5	2.1–10	7 (0.8)
Enzalutamide	4.5	2.0–10	6 (0.7)
Influenza vaccine	2.9	1.9–4.0	23 (2.7)

a One “unspecified” case was reported (Supplementary Table S1- Reported suspected drugs in patients with TGA).

### Disproportionality Analysis

The disproportionality analysis highlighted 18 active ingredients subject to potential safety signals regarding TGA (IC025 > 0), ranked in Table 2.

Among them, COVID-19 vaccines were characterized by a significant ROR (ROR 5.1; 95%CI 4.4–6.0). Tozinameran reached the strongest ROR (ROR 4.6; 95%CI 3.9–5.0), followed by elasomeran (ROR 4.4; 95%CI 3.4–6.0), AZD1222 (ROR 3.8; 95%CI 3.0–5.0) and JNJ-78436735 (ROR 3.7; 95%CI 2.1–6.0). In addition, our subgroup analysis for COVID-19 vaccines also showed disproportionate reporting for all sex and age categories, except the 18–44 years old. A significant ROR (ROR 5.6; 95%CI 4.1–7.8) was also found for all physician-reported cases related to COVID-19 vaccines. These analyses are displayed in Table 3. Furthermore, influenza vaccines also had a significant ROR (ROR 2.9; 95%CI 1.9–4.0) (Table 2).

**TABLE 3:** Subgroup disproportionality analyses for COVID-19 vaccine reports of TGA.

Characteristics	ROR	95% CI	IC025
Sex			
Female	5.0	4.2–6.0	1.6
Male	5.2	4.1–6.5	1.7
Age			
18–44 years	1.7	0.8–3.6	-0.6
45–64 years	4.2	3.3–5.4	1.3
65–74 years	7.3	5.7–9.4	2.0
≥75 years	7.9	5.3–11.9	1.9
Reporter			
Physician	5.6	4.1–7.6	1.8

PDE5 inhibitors, tadalafil and sildenafil, yielded RORs of 19.3 (95%CI 11.9–31.0) and 9.9 (95%CI 6.1–16.0), respectively. RORs for statins were 11.1 (95%CI 8.1–15.0) for atorvastatin, 8.1 (95%CI 5.1–13.0) for simvastatin and 8.1 (95%CI 4.9–15.0) for rosuvastatin. In addition, a ROR of 12.2 (95%CI 7.5–20) was found for zolpidem.

## Discussion

We show that COVID-19 vaccines are, by far, the most reported drugs associated with TGAs in the WHO pharmacovigilance database, representing more than one-third of the cases. The disproportionality analysis suggests a potential pharmacovigilance signal. Even if the signal appears fairly homogeneous between the different COVID-19 vaccines, mRNA vaccines tozinameran and elasomeran stand out in terms of disproportionality. Despite rigorous monitoring of ADRs associated with COVID-19 vaccines, TGAs seem to have escaped scrutiny so far, even if a few cases had already been singled out (Koh et al., 2021).

Former studies found that TGAs mostly occur in the elderly population. (Bartsch and Deuschl, 2010; Arena and Rabinstein, 2015). Age accumulates vascular risk factors and predisposes the patient to cerebral inflammation. Our analysis of VigiBase^®^ showed a foreseeable age distribution in all cases, as well as in COVID-19 vaccine cases. Most COVID-19 vaccine-related TGAs involved women. It may reflect the fact that most spontaneous reports of serious COVID-19 vaccine-associated ADRs in VigiBase^®^ originate from women. (Dutta et al., 2021).

TGA has been ascribed, among others, to temporary alterations of the hippocampus. (Bartsch et al., 2006). Consequently, COVID-19 vaccination, underpinned by metabolic stress, could result in cerebrovascular and inflammatory manifestations. Thus, migraine, especially when vaccine-associated, (Göbel et al., 2021), may lead to an increased incidence of TGA. (Ding and Peng, 2020). The psychological impact of vaccination itself, as a factor of sudden change and anxiety, may contribute to the occurrence of TGA following COVID-19 vaccination. (Inzitari, 2005). In addition, during the pandemic, COVID-19 infection itself, (Werner et al., 2020; Hornick et al., 2022), increased stress induced by social distancing, uncertainty concerning the future, and the fear of getting infected could trigger TGA. (Ramanathan and Wachsman, 2021). Interestingly, the potential role of the influenza vaccine in TGA has not yet been studied. A drug inflammatory class effect with vaccines could be evoked.

Furthermore, in terms of the number of cases and strength of the association, three other drug classes stood out, PDE5 inhibitors, statins, and zolpidem. This result was somehow expected, as their respective labelings mention amnesia (which covers a broader spectrum of affections than TGA taken in isolation). (FDA, 2008; FDA, 2011; FDA, 2012).

This study is the first to rely on a global analysis of TGA reports from the WHO safety database. Nonetheless, its limitations are inherent to postmarketing pharmacovigilance studies, including reporting bias, under-reporting, coding heterogeneity, and incomplete data. Further, only 852 cases of TGA were recorded in VigiBase^®^ since 1967 (first reports in the database), which suggests a very significant under-reporting. Our subgroup analysis in COVID-19 vaccines cases displayed disproportionate reporting for both sexes, and all age ranges except the 18–44 years one. While the latter may be explained by a lack of power (9 patients in this range), the significant disproportionality in all other groups strengthens our primary approach.

A negative COVID-19 serostatus of the patients before vaccination, paramount to evoke the causality of the vaccines, is unfortunately lacking. Due to potential misdiagnosis of other neurological affections, differential diagnosis may be missing in some cases. However, we aimed to improve the accuracy of our results regarding the liability of TGA diagnosis. Our sensitivity analysis conducted among COVID-19 vaccines-related TGA cases that were reported by physicians corroborates our findings. In addition, some authors (Larner, 2017) suggested a revision of the Hodges and Warlow diagnostic criteria for TGA, with an inclusion of sequential diffusion-weighted magnetic resonance imaging findings. Further, a better harmonization of the diagnosis and coding of drug-induced TGA would pave the way to more homogeneous patient cohorts, of particular interest when studying TGA.

Despite an increased rate of reporting with COVID-19 vaccines and the COVID-19-associated confounders, the observed disproportionality suggests a statistically significant pharmacovigilance signal. Indeed, the Weber effect mostly concerns tozinameran, which is by far the most administered COVID-19 vaccine in United States and in Europe. Yet, the disproportionality approach indicates that TGAs stand out among other COVID-19 vaccines’ ADRs. In any case, pharmacovigilance studies aim to raise awareness by suggesting potential drug signals. While these findings do not call into question the benefit/risk ratio of COVID-19 vaccination, their causal association with TGA must be further assessed.

## Data Availability

The data that support the findings of this study are available from Uppsala Monitoring Center (UMC) but restrictions apply to the availability of these data, which were used under license for the current study, and so are not publicly available. Access to VigiBase® is available without fees to FR. Data are however available from the authors upon reasonable request and with permission of UMC.
