# Supplementary material for: COVID-19 Vaccine-Associated Transient Global Amnesia: A Disproportionality Analysis of the WHO Safety Database
Source: Front Pharmacol. 2022 May 20;13:909412. doi: 10.3389/fphar.2022.909412 (PMC9164011; doi:10.3389/fphar.2022.909412)
Supplement: Supplementary file 1 [file Table1.DOCX]

Supplementary Material

**Table S1.** Reported suspected drugs in patients with TGA

| **Drugs** | **Number (%)** |
| --- | --- |
| **COVID-19 vaccine** | **289 (33.9)** |
| Tozinameran | 147 (50.8) |
| ChAdOx1 nCoV-19 AZD1222 | 69 (23.8) |
| Elasomeran | 60 (20.8) |
| JNJ 78436735 | 12 (4.2) |
| Unspecified | 1 (0.3) |
| Atorvastatin | 41 (4.8) |
| Influenza vaccine | 23 (2.7) |
| Simvastatin | 18 (2.1) |
| Sildenafil | 17 (2.0) |
| Tadalafil | 17 (2.0) |
| Rosuvastatin | 15 (1.8) |
| Zolpidem | 15 (1.8) |
| Alendronic acid | 11 (1.3) |
| Tramadol | 9 (1.1) |
| Lenalidomide | 9 (1.1) |
| Etanercept | 8 (0.9) |
| Baclofen | 7 (0.8) |
| Heparin | 7 (0.8) |
| Varicella zoster vaccine | 7 (0.8) |
| Vardenafil | 6 (0.7) |
| Enzalutamide | 6 (0.7) |
| Levothyroxine | 5 (0.6) |
| Denosumab | 5 (0.6) |
| Pneumococcal vaccine | 5 (0.6) |
| Methyl aminolevulinate | 5 (0.6) |
| Prednisone | 4 (0.5) |
| Methotrexate | 4 (0.5) |
| Fluoxetine | 4 (0.5) |
| Fampridine | 4 (0.5) |
| Bupropion | 4 (0.5) |
| Ibandronic acid | 4 (0.5) |
| Etoricoxib | 4 (0.5) |
| Dobutamine | 4 (0.5) |
| Fumaric acid | 4 (0.5) |
| Iodixanol | 4 (0.5) |
| Iomeprol | 4 (0.5) |
| Duloxetine | 4 (0.5) |
| Natalizumab | 4 (0.5) |
| Ibrutinib | 4 (0.5) |
